# Drug-Coated Balloon vs. Stent for *de novo* Non-small Coronary Artery Disease: A Systematic Review and Meta-Analysis

**DOI:** 10.3389/fcvm.2021.700235

**Published:** 2021-12-10

**Authors:** Kaiwen Sun, Zhenzhu Liu, Hongyan Wang

**Affiliations:** ^1^The Second Hospital of Dalian Medical University, Dalian Medical University, Dalian, China; ^2^Department of Cardiovascular Medicine, The Second Hospital of Dalian Medical University, Dalian, China

**Keywords:** drug-coated balloon, *de novo* vessels, non-small, stent, systematic review

## Abstract

**Introduction:** Drug-coated balloon (DCB) has been an attractive option in *de novo* vessels. A systematic review and meta-analysis were conducted to evaluate the efficacy and safety of DCB vs. stent for treating *de novo* lesions in non-small vessels.

**Methods:** Studies in PubMed, Embase, the Cochrane Central Register of Controlled Trials, and Web of Science were searched (from their commencement to March 2021). This meta-analysis was performed by Review Manager 5.3.

**Results:** A total of 3 random controlled trials (RCTs) with 255 patients and 2 observational studies (OS) with 265 patients were included in this meta-analysis following our inclusion criteria. It could be observed that DCB presented no significant difference in cardiac death (CD) (*RR* 0.33, 95% CI [0.01, 8.29], *p* = 0.50 in OS), myocardial infarction (MI) (*RR* 0.49, 95% CI [0.09, 2.50], *p* = 0.39 in RCT), target lesion revascularization (TLR) (*RR* 0.64, 95% CI [0.19, 2.18], *p* = 0.47 in RCT) (*RR* 1.72, 95% CI [0.56, 5.26], *p* = 0.34 in OS), and late lumen loss (LLL) (SMD −0.48, 95% CI [−1.32, 0.36], *p* = 0.26 in RCT) for *de novo* non-small coronary artery disease (CAD) compared with stents, whereas minimal lumen diameter (MLD) including MLD1 (SMD −0.67, 95% CI [−0.92 −0.42], *p* < 0.00001 in RCT) and MLD2 (SMD −0.36, 95% CI [−0.61 −0.11], *p* = 0.004 in RCT) was smaller in DCB group.

**Conclusion:** This systematic review showed that DCB might provide a promising way on *de novo* non-small coronary artery disease compared with stents. However, more RCTs are still needed to further prove the benefits of the DCB strategy.

**Systematic Review Registration:** https://www.crd.york.ac.uk/PROSPERO/#recordDetails.

## Introduction

Coronary artery disease (CAD) is still the leading cause of human death and disability worldwide according to the latest epidemiological data from the Global Burden of Disease (GBD) (1). Percutaneous coronary intervention (PCI) is a therapeutic intervention by expanding the stenosis or occlusion of the vascular lumen, which improves the circulation of myocardium. With the development of technology, PCI has been applied to more complex lesions among patients with CAD (2, 3). At present, most of the PCI is expanding the target lesions with stents, from bare metal stent (BMS) to first-generation drug-eluting stent (DES) and second- or newer-generation DES, which can not only significantly reduce the incidence and mortality of myocardial infarction (MI) in high-risk patients with massive myocardial ischemia, but also improve the quality of life of patients. Despite the superiority of stents in the management of CAD, very late adverse cardiovascular events still exist after stent implantation (4). Though with the advent and application of DES, complications such as in-stent restenosis (ISR) and stent thrombosis still occur, leading to the recurrence of angina or acute coronary syndrome (ACS) (5, 6). There are also technical challenges in the application of stents in complex lesions, which affect its long-term efficacy. A recent study reported a new complication of longitudinal stent deformation (LSD), which is defined as the distortion or shortening of intracoronary stents after successful stent deployment because of the reduced longitudinal strength of stents (7). There are also some rare complications such as spontaneous coronary artery pseudo aneurysm and infection according to the report (8, 9).

In recent years, drug-coated balloon (DCB) has been investigated and applied in clinical for its no residues. It has become 1 of the preferred options for the treatment of ISR by the ESC guideline (10). There are also many potential benefits such as *de novo* vessel disease and patients with a high risk of bleeding. In addition, there are other potential indications, such as bifurcation lesions, chronic total occlusion lesions, and others (11–13). With the advantage of DCB, there are some systematic reviews and meta-analysis based on the role of DCB in *de novo* vessels, whose results showed that DCB was noninferior to the stent strategy when treating the target lesions (14–16). However, those results were concentrated more on *de novo* small vessels or unclassified vessel diameter of *de novo* vessels. Besides, those results included BMS as a control, which has been rarely applied and not recommended for PCI by the recent guidelines. With the development and advance of DCB, there were several studies that used DCB for treating with *de novo* non-small vessels (17–21). Lin et al. (22) did a meta-analysis in *de novo* large vessels, though remained some limitations. First, only 1 study with stable CAD was included in their study and the subgroup analysis was limited. Second, the end points in their study were not enough since there were more clinical and angiographic outcomes which could be used to assess the efficacy and safety of DCB. Finally, the number of studies was not enough and contained 1 nonrandom controlled trial (RCT).

Therefore, we conducted a systematic review and meta-analysis to evaluate the efficacy and safety of DCB vs. stent for treating *de novo* lesions in non-small vessels.

## Materials and Methods

### Protocol

This meta-analysis followed the Preferred Reporting Items for Systematic Meta-Analysis (PRISMA) statement (23). The protocol was registered at PROSPERO (registration number: CRD42021244832).

### Search Strategy

The following electronic databases were searched: PubMed, Embase, the Cochrane Central Register of Controlled Trials, and Web of Science from their commencement to March 2021 for the relevant literature. A manual search of references was performed for additional omitted studies. The specific search strategies were presented in the supplemental.

### Selection Criteria

The literature was independently screened and evaluated by 2 authors (Sun and Liu). In total, 2 authors scanned the title and abstract according to the inclusion criteria and then screened again by reading the full text. If there were any disagreements, it would be discussed with another author (Wang) and resolved by consensus, or contacted the original author of the article if necessary.

The inclusion criteria were as follows: (1) patients with CAD; (2) PCI for *de novo* lesions with lumen diameters ≥ 2.5 mm; (3) DCB as experimental group without any restriction on the type of DCB; (4) stent as control group for DES only; and (5) studies included RCT and observational study (OS).

The exclusion criteria were as follows: (1) patients with peripheral vascular diseases; (2) PCI for *de novo* lesions with lumen diameters < 2.5 mm; (3) studies were reviewed or conference abstract; and (4) data inefficient.

### Study Outcomes and Definition

The study outcomes could be divided into clinical outcomes and angiographic outcomes. The clinical outcomes included cardiac death (CD), MI, and target lesion revascularization (TLR). CD was defined as death caused by cardiac factors. MI was defined based on the latest ESC guidelines (10). TLR was defined as any repeat revascularization due to restenosis in treated segments including 5 mm proximally and distally. The angiographic outcomes included minimal lumen diameter (MLD), which was divided into MLD1 (MLD immediately after PCI) and MLD2 (MLD at follow-up angiography), and late lumen loss (LLL), which means MLD1 minus MLD2. The angiographic outcomes were obtained by quantitative coronary angiography (QCA).

### Data Extraction

The following data were extracted from eligible studies independently by 2 authors (Sun and Liu): the study of the author, year, and country; patients' indication; the number of patients, age, and sex in each group; the vessel diameter and length; the main characteristics of DCB; the type of DCB and stents; lesion preparation for DCB and stents; clinical and angiographic outcomes and follow-ups.

### Quality Assessment

The quality of the eligible RCT studies and the risk of bias were assessed by 2 independent authors (Sun and Liu) using the Cochrane Handbook for Systematic Reviews of Interventions, version 5.1.0, which contains seven criteria: random sequence generation, allocation concealment, blinding of participants and personnel, blinding of outcome assessment, incomplete outcome data, selective outcome reporting, and other bias. These seven criteria were rated as “low risk,” “unclear risk,” or “high risk” depending on the characteristics of each criterion reported in the study. For OS, the quality of the studies was assessed by the Newcastle–Ottawa Scale (NOS) with 8 items including selection, comparability, and outcome. A study with more than 6 stars was regarded as a high-quality one.

### Statistical Analysis

The effect of DCB or stents for *de novo* non-small vessels was measured by relative risks (*RR*) for all clinical outcomes and standard mean difference (SMD) for all angiographic outcomes. As several included studies did not offer the changes in the standard deviation (SD) of LLL or MLD from the baseline values, we calculated the SD change by the following formula (1). The coefficient was taken as 0.5 according to other eligible studies that provided the SD baseline, SD final, and SD change.

SD change = √[(SD pretreatment) ^2^ + (SD posttreatment) ^2^ - (2^*^ coefficient ^*^SD pretreatment ^*^SD posttreatment)] (1)

This meta-analysis was performed by Review Manager 5.3 (Cochrane Collaboration, 2014). Heterogeneity of the studies was assessed by the Cochran's Q statistic and *I*^2^ test. Studies were considered homogeneous if the *p*-value of the Q-test was > 0.1 or the *I*^2^ value was <50%, or it would be considered as heterogeneity. A random effect model was chosen to analyze the data.

To further explore the other factors associated with the results, a series of subgroup analyses were performed by Review Manager 5.3 (Cochrane Collaboration, 2014), including the type of patient status, age, the type of DCB, and the clinical or angiographic follow-up, with *p* < 0.05 considered statistically significant. The sensitivity analysis was done using the leave-one-out method to examine the stability of the results.

## Results

### Description of Studies

**Figure 1:**
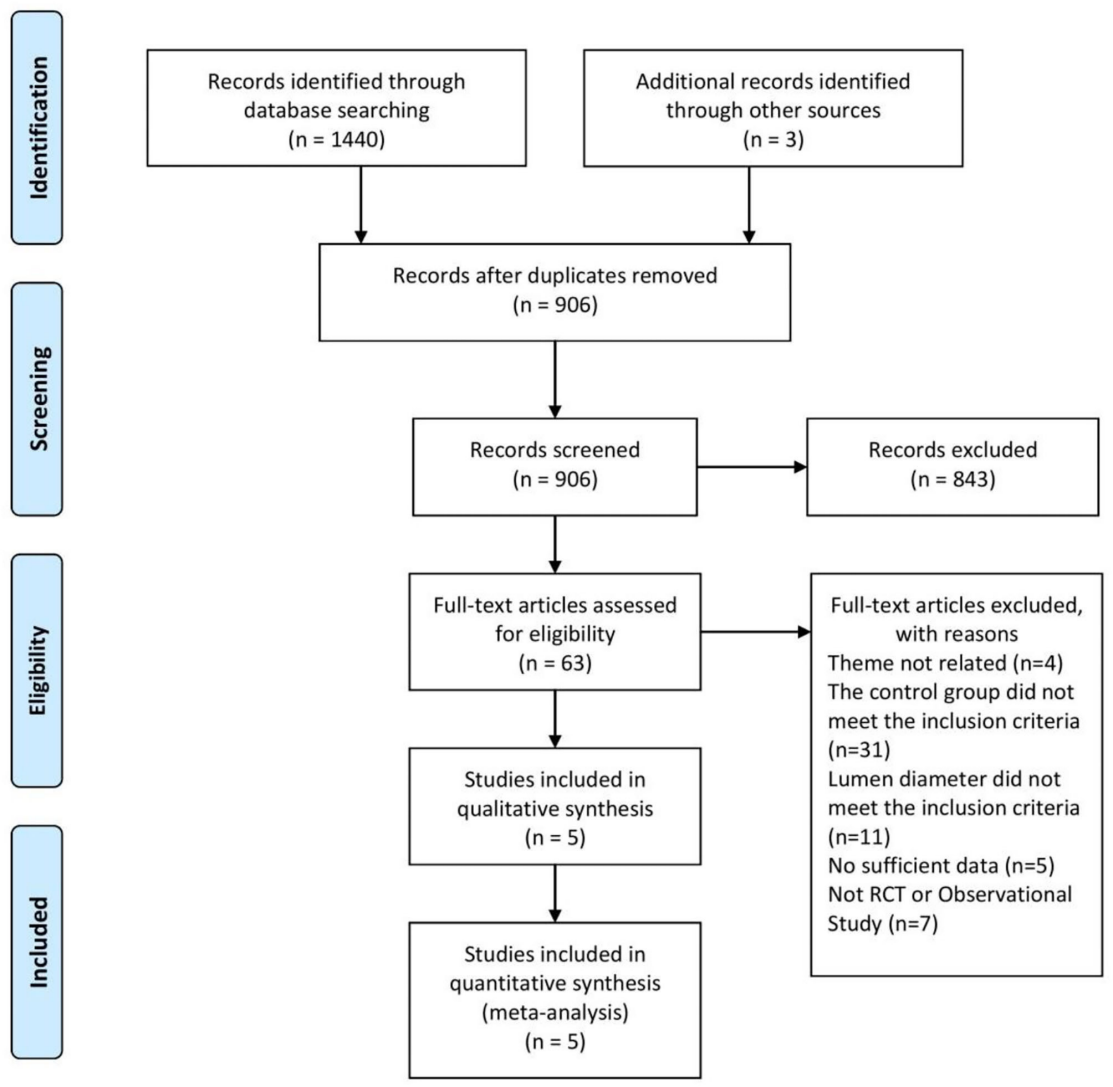
Study selection process.

A total of 1,443 studies were retrieved based on the search terms and strategies described ahead. There had been 906 studies to be screened by titles and abstracts since 843 studies were removed because of repetition. Of the 906 studies, 63 studies remained to be read the full text for further exclusion. Finally, 5 studies (17–21), including 3 RCTs and 2 OS, were selected and included in this meta-analysis with the inclusion criteria (Figure 1). The characteristics of included study were summarized in the Table 1 as shown below.

**Table 1:** The characteristics of the study included.

**Author**	**Year**	**Country**	**Type of study**	**Patients' indications**	**Number of patient (DCB/Control)**	**Age (years) (DCB/Control)**	**Sex (male/female)**	**Vessel diameter(mm) (DCB/Control)**	**Vessel length(mm) (DCB/Control)**	**The main characteristics of DCB**	**Type of DCB**	**Type of control**	**Lesion preparation for DCB**	**Lesion preparation for Control**	**Clinical outcomes**	**Angiographic outcomes**	**Clinical follow-up (months)**	**Angiographic follow-up (months)**
Gobić et al. (18)	2017	Croatia	RCT	STEMI	38/37	56.6 ± 13.2 54.3 ± 10.6	56/22	2.50-4.00	NA	Paclitaxel-coated balloon	DCB:SeQuent Please	DES: Biomime	Thrombus aspiration and balloon dilation	Thrombus aspiration or balloon dilation or both	CD MI TLR	MLD LLL	6	6
Nishiyama et al. (17)	2016	Japan	RCT	CCAD	27/33	67.3 ± 11.1 70.6 ± 9.0	44/16	2.88 ± 0.57 2.72 ± 0.64	16.13 ± 5.25 18.14 ± 7.41	Paclitaxel-coated balloon	DCB:SeQuent Please	DES: Xience	Nonslip element balloon	NA	CD MI TLR	MLD LLL	8	8
Vos et al. (19)	2019	Netherlands	RCT	STEMI	60/60	57.4 ± 9.2 57.3 ± 8.3	104/16	3.28 ± 0.52 3.20 ± 0.48	NA	Paclitaxel-coated balloon	DCB: Pantera Lux	DES:Orsiro/Xience	Thrombus aspiration and balloon dilation	NA	CD MI TLR	MLD LLL	9	9
Her et al. (20)	2018	South Korea	OS	CCAD	54/54	57.6 ± 9.5 58.9 ± 10.4	28/80	2.70 ± 0.40 2.80 ± 0.40	19.90 ± 5.20 21.20 ± 5.90	Paclitaxel-coated balloon	DCB:SeQuent Please	DES: Xience prime and Xience xpedition / Resolute integrity /Biomatrix Flex/ Orsiro/ Promus Premier	The standard balloon	NA	CD MI TLR	NA	12	12
Iwasaki et al. (21)	2020	Japan	OS	Calcified Lesions	69/88	76.0 ± 7.2 74.0 ± 8.4	102/55	2.97 ± 0.45 3.03 ± 0.36	19.00 ± 11.00 19.00 ± 10.00	Paclitaxel-coated balloon	DCB:SeQuent Please	DES:Xience/Synergy/ Nobori/Ultimaster/ Resolute Onyx/ Biosensors Interventional Technologies	Rotational atherectomy	Rotational atherectomy	CD MI TLR	MLD LLL	12	12

*RCT, randomized controlled trial; OS, observational study; STEMI, ST-segment elevation myocardial infarction; CCAD, chronic coronary artery disease; NA, not available; DCB, drug-coated balloon; DES, drug-eluting stent; CD, cardiac death; MI, myocardial infarction; TLR, target lesion revascularization; MLD, minimal lumen diameter; LLL, late lumen loss*.

### Quality Assessment

**Figure 2:**
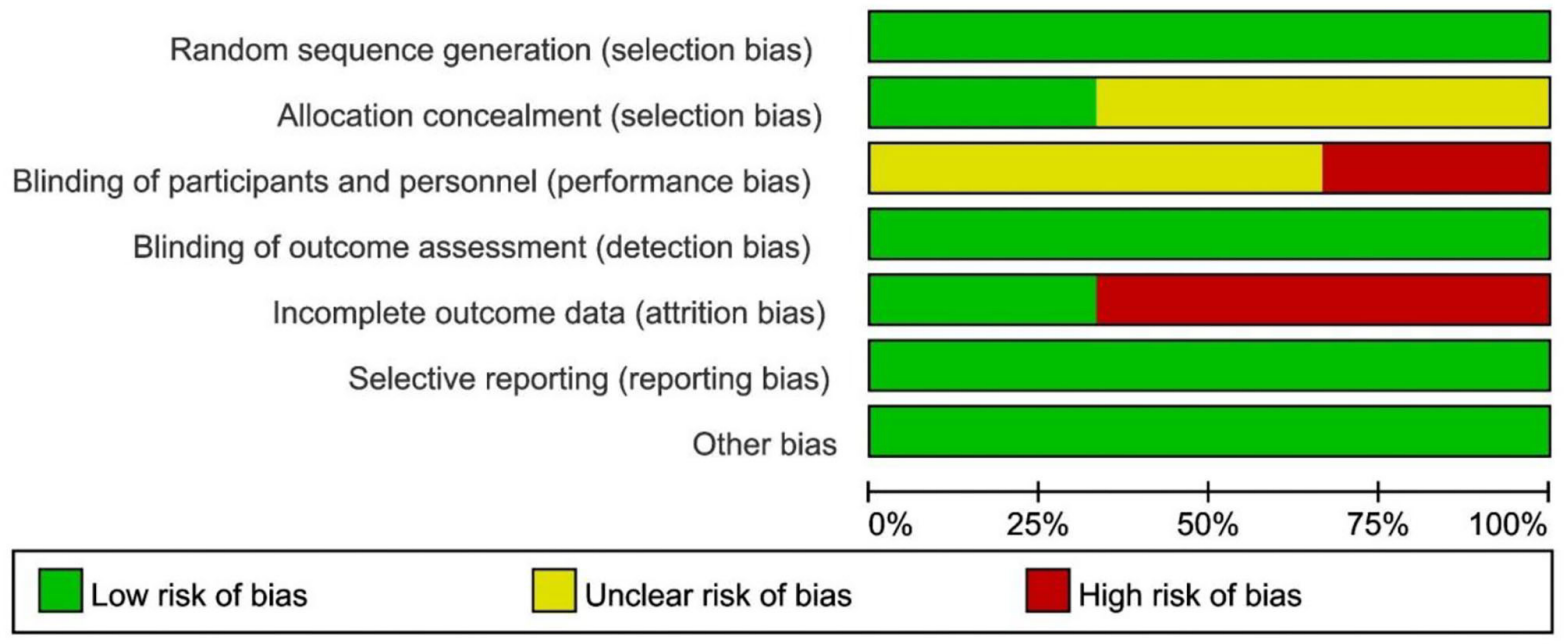
Risk of bias graph review authors' judgments about each risk of bias item presented as percentages across all included RCT.

**Figure 3:**
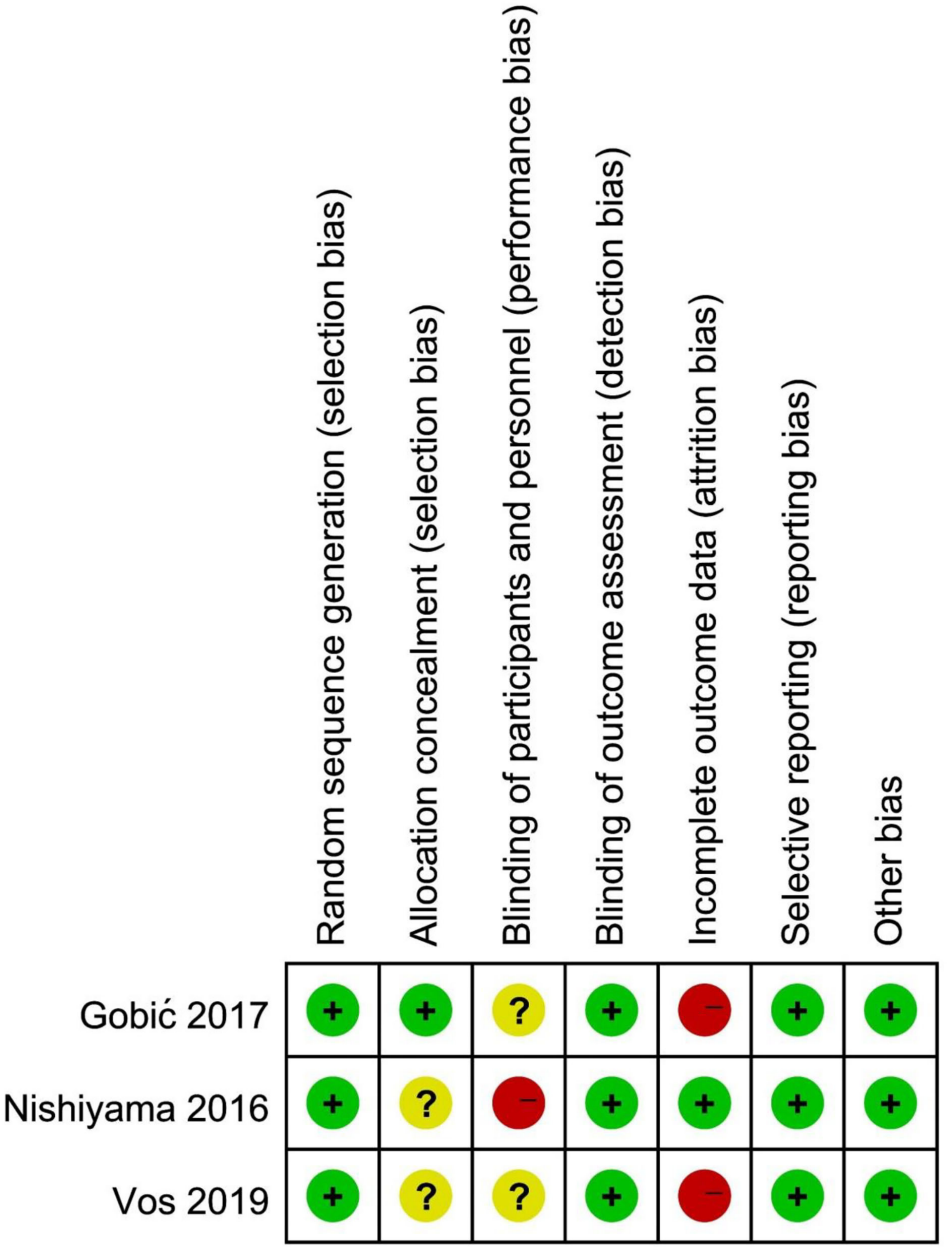
Risk of bias summary review authors' judgements about each risk of bias item for each included RCT.

The Cochrane risk of bias tool was used to assess the methodological quality of the included RCT studies, as were presented in the Figures 2, 3. For selection bias, most studies showed a low risk whereas 2 studies (17, 19) did not specifically describe allocation concealment. For performance bias, 1 study (17) did not take any blind methods, which might have an effect on the results. For detection bias, all studies could explain the results despite the blind method. In terms of attrition bias and reporting bias, 2 studies (18, 19) failed to provide comprehensive information on follow-up or exclusion. For reporting bias and other bias, no study presented any obvious one. The NOS was used to assess the quality of OS from selection, comparability, and outcome. Both the 2 OS were of a high quality by rewarding 8 and 7 stars which were shown in Table 2.

**Table 2:** Study quality of OS.

**Author**	**Year**	**Representativeness of the exposed cohort**	**Selection of the nonexposed cohort**	**Ascertainment of exposure**	**Demonstration that outcome of interest was not present at the start of study**	**Comparability of cohorts on the basis of the design or analysis**	**Assessment of outcome**	**Was follow up long enough for outcomes to occur**	**Adequacy of follow-up of cohorts**	**Quality score**
Her et al. (20)	2018	*	*	*	*	*	*	*	*	8
Iwasaki et al. (21)	2020	*	*	*	*	*	*	*		7

### Clinical and Angiographic Outcomes

#### CD and MI

**Figure 4:**
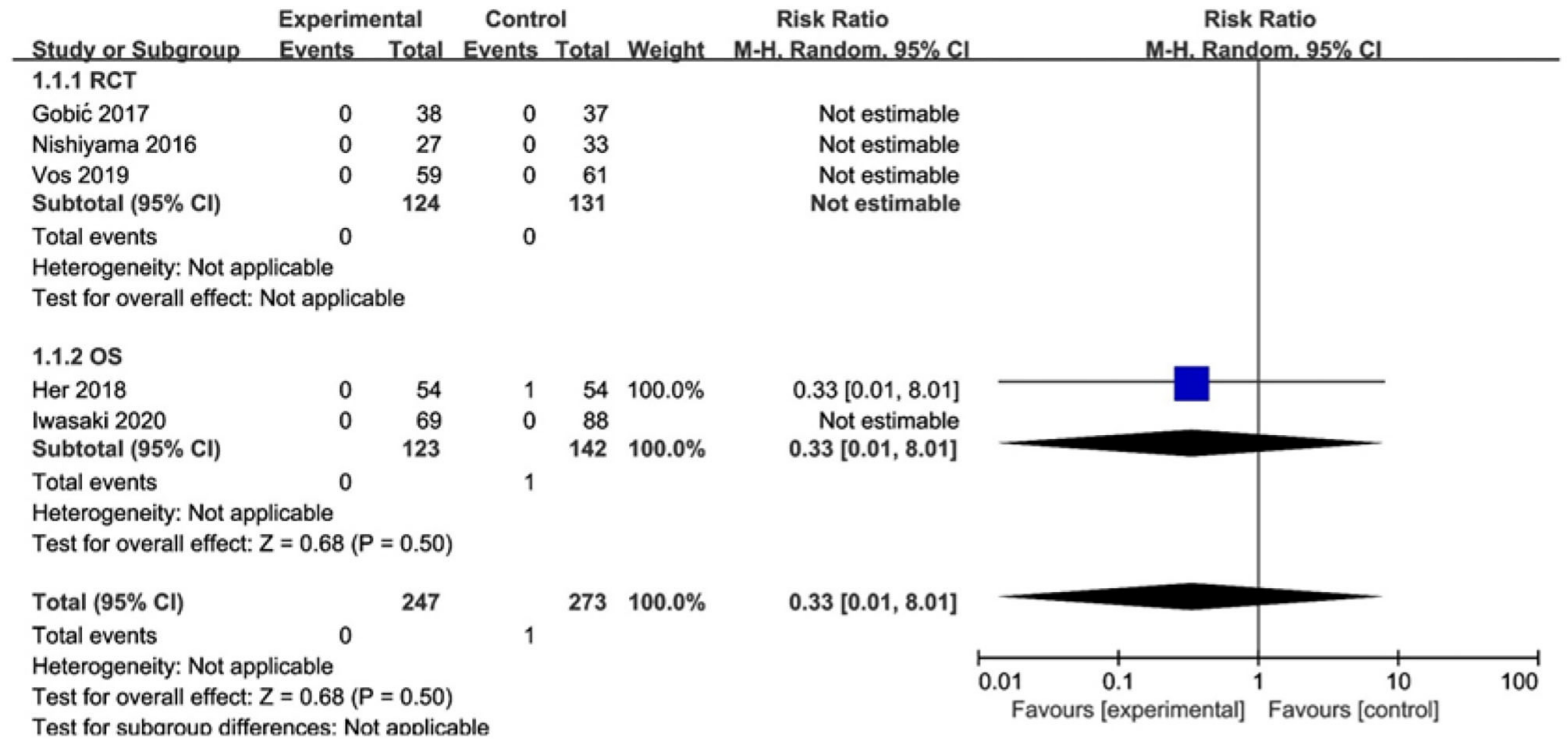
Meta-analysis of the effects of DCB compared with control in CD.

**Figure 5:**
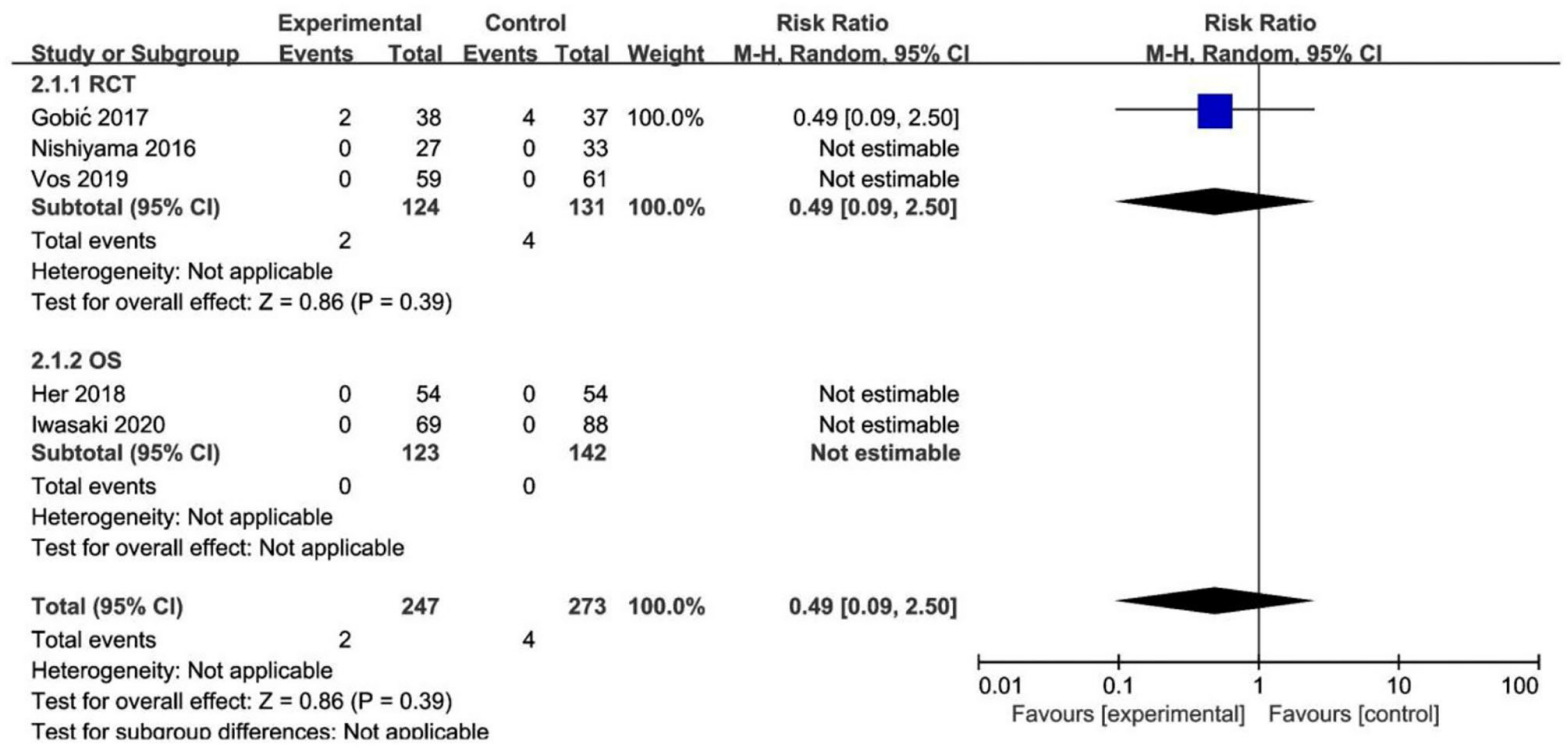
Meta-analysis of the effects of DCB compared with control in MI.

Of the 3 RCTs and 2 OS, CD was observed in only 1 study (20), with no significant difference between the DCB and stents (*RR* 0.33, 95% CI [0.01, 8.29], *p* = 0.50). MI was observed in only 1 study (18), with no significant difference between the DCB and stents (*RR* 0.49, 95% CI [0.09, 2.50], *p* = 0.39) either. The specific description results were presented in the Figures 4, 5.

#### TLR

**Figure 6:**
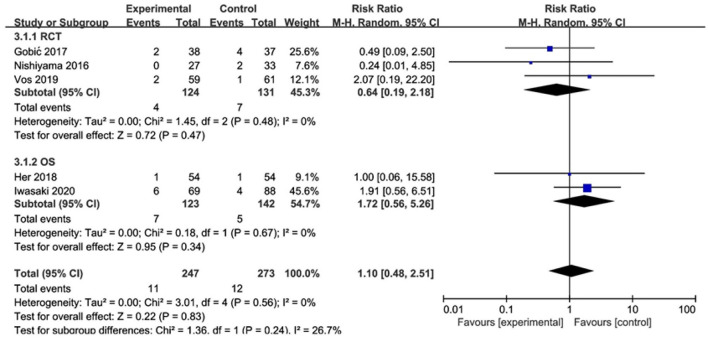
Meta-analysis of the effects of DCB compared with control in TLR.

In RCT, the results showed no significant difference between DCB and stents in the rate of TLR (*RR* 0.64, 95% CI [0.19, 2.18], *p* = 0.47). In OS, there was no significant difference in the rate of TLR (*RR* 1.72, 95% CI [0.56, 5.26], *p* = 0.34) either. There was lower heterogeneity in RCT (*I*^2^ = 0, *p* = 0.48) and OS (*I*^2^ = 0, *p* = 0.67). The specific description results were presented in the Figure 6.

#### LLL

**Figure 7:**
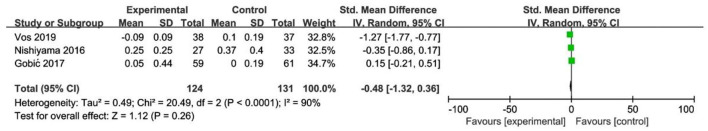
Meta-analysis of the effects of DCB compared with control in LLL.

In RCT, the results showed no significant difference between DCB and stents with significant heterogeneity (SMD −0.48, 95% CI [−1.32, 0.36], *p* = 0.26, *I*^2^ = 90%, *p* < 0.0001). In OS, only 1 study (21) provided the angiographic outcomes, which could not be calculated the combined effect size (CES) and thus it was excluded. The specific description results were presented in the Figure 7.

#### MLD1

**Figure 8:**
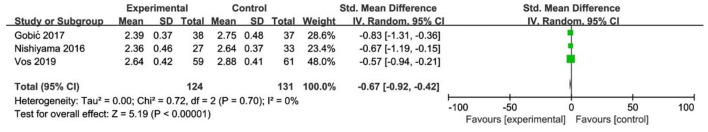
Meta-analysis of the effects of DCB compared with control in MLD1.

In RCT, the results showed that MLD1 in DCB group was smaller than stent group with lower heterogeneity (SMD −0.67, 95% CI [−0.92 −0.42], *p* < 0.00001, *I*^2^ = 0, *p* = 0.70). In OS, only 1 study (21) provided the angiographic outcomes, which could not be calculated the CES and thus it was excluded. The specific description results were presented in the Figure 8.

#### MLD2

**Figure 9:**
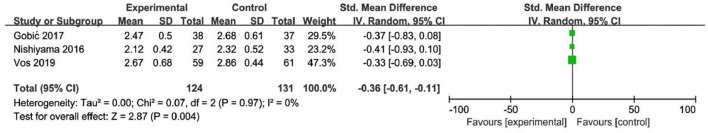
Meta-analysis of the effects of DCB compared with control in MLD2.

In RCT, the results showed that MLD2 in DCB group was smaller than stent group with lower heterogeneity (SMD −0.36, 95% CI [−0.61 −0.11], *p* = 0.004, *I*^2^ = 0, *p* = 0.97). In OS, only 1 study (21) provided the angiographic outcomes, which could not be calculated the CES and thus it was excluded. The specific description results were presented in the Figure 9.

### Subgroup and Sensitivity Analysis

Subgroup analyses were performed to evaluate the effect of type of patient status, age, the type of DCB, and the clinical or angiographic follow-up in TLR, LLL, and MLD. For TLR, there was no significant difference between DCB and stents in the subgroups. For LLL, there was still a high heterogeneity in most subgroups though the heterogeneity was lower when the follow-up was more than 6 months and the results showed no significant difference. For MLD, it could be observed that MLD1 and MLD2 in DCB group were smaller in each subgroup. The detailed description results were shown in the form below (Tables 3–6).

**Table 3:** The subgroup analysis of TLR.

	**Group**	**The number**	***RR*** **(95% CI)**	***p*** **-value**	***I*** **^2^%**
		**of study**	**(95% CI)**	
The type of	CCAD	1	0.24[0.01,4.85]	0.35	NA
patient status	STEMI	2	0.78[0.20,2.98]	0.71	0
Age	≤ 60 years old	2	0.78[0.20,2.98]	0.71	0
	> 60 years old	1	0.24[0.01,4.85]	0.35	NA
The type of DCB	SeQuent Please	2	0.41[0.10,1.74]	0.23	0
	Pantera Lux	1	2.07[0.19,22.20]	0.55	NA
Follow-up	≤ 6 months	1	0.49[0.09,2.50]	0.23	NA
	> 6 months	2	0.87[0.11,6.91]	0.89	18%

**Table 4:** The subgroup analysis of LLL.

	**Group**	**The number**	**SMD (95% CI)**	***p*** **-value**	***I*** **^2^%**
		**of study**	**(95% CI)**		
The type of	CCAD	1	−0.35[−0.86,0.17]	0.18	NA
patient status	STEMI	2	−0.55[−1.94,0.84]	0.44	95%
Age	≤ 60 years old	2	−0.55[−1.94,0.84]	0.44	95%
	>60 years old	1	−0.35[−0.86,0.17]	0.18	NA
The type	SeQuent Please	2	−0.81[−1.72,0.09]	0.08	84%
of DCB	Pantera Lux	1	0.15[−0.21,0.51]	0.42	NA
Follow–up	≤ 6 months	1	−1.27[−1.77,−0.77]	<0.00001	NA
	>6 months	2	−0.48[−1.32,0.36]	0.79	58%

**Table 5:** The subgroup analysis of MLD1.

	**Group**	**The number**	**Mean ± SD**	***p*** **-value**	***I*** **^2^%**
		**of study**	**(95% CI)**		
The type of	CCAD	1	−0.67[−1.19,−0.15]	0.01	NA
patient status	STEMI	2	−0.67[−0.96,−0.38]	<0.00001	0
Age	≤ 60 years old	2	−0.67[−0.96,−0.38]	<0.00001	0
	>60 years old	1	−0.67[−1.19,−0.15]	0.01	NA
The type	SeQuent Please	2	−0.76[−1.11,−0.41]	<0.0001	0
of DCB	Pantera Lux	1	−0.57[−0.94,−0.21]	0.002	NA

**Table 6:** The subgroup analysis of MLD2.

	**Group**	**The number**	**Mean ± SD**	***p*** **-value**	***I*** **^2^%**
		**of study**	**(95% CI)**		
The type of	CCAD	1	−0.41[−0.93,0.10]	0.12	NA
patient status	STEMI	2	−0.35[−0.63,−0.06]	0.02	0
Age	≤ 60 years old	2	−0.35[−0.63,−0.06]	0.02	0
	>60 years old	1	−0.41[−0.93,0.10]	0.12	NA
The type	SeQuent Please	2	−0.39[−0.73,−0.05]	0.02	0
of DCB	Pantera Lux	1	−0.33[−0.69,0.03]	0.07	NA
Follow-up	≤ 6 months	1	−0.37[−0.83,0.08]	0.11	NA
	>6 months	2	−0.36[−0.65,−0.06]	0.02	0

To explore the impact of each study on the stability of the combined results, a sensitivity analysis was conducted using the one-study removed approach. The heterogeneity and the CES of TLR (*RR* 0.64, 95% CI [0.19, 2.18]), MLD1 (SMD −0.67, 95% CI [−0.92 −0.42]), and MLD2 (SMD −0.36, 95% CI [−0.61 −0.11]) were not significantly changed, which indicated the results were robust enough. We observed that the heterogeneity of LLL was declined (*I*^2^ from 90% turned to 58%) when the study of Gobic et al. (18) was removed, whereas the CES (SMD −0.48, 95% CI [−1.32, 0.36]) was not significantly changed.

## Discussion

This systematic review and meta-analysis included 3 RCTs and 2 OS following the inclusion criteria. The results indicated that DCB presented no significant difference in CD, MI, TLR, and LLL for *de novo* non-small CAD compared with stents, whereas MLD1 and MLD2 were smaller in DCB group. We performed the subgroup analysis, which could be observed that MLD1 and MLD2 were also smaller. Thus, these data indicated that DCB might provide a promising way for *de novo* non-small CAD.

There had been studies on the comparison between DCB and DES or other types of stents, but their data only focused on the small vessels or did not consider the vessel diameter of *de novo* lesions. Lin et al. (22)suggested that DCB appeared to be an interventional and stentless alternative for treating *de novo* coronary lesions in large vessels, though there were still some limitations left as described before. Thus, this systematic review and meta-analysis were conducted to evaluate DCB vs. stent for treating *de novo* lesions in non-small vessels. Before the implantation of DCB or stent, lesion preparation was introduced to improve the procedure success rate and reduce late adverse events. Balloon dilation is the most common way and has been widely applied for lesion preparation. Patients with STEMI would be considered adding for thrombus aspiration. However, it remains potential complications including suboptimal dilatation, dissections, plaque shift, elastic recoil, and vessel perforation with high-pressure balloon inflation (24). Iwasaki et al. (21) chose rotational atherectomy which has been demonstrated a preferred choice in calcified lesions in both DCB and stent group for balloon or stent delivery and expansion, as it could reduce calcified plaque. For DCB, lesion preparation might be more important without stent implantations due to elastic recoil and dissections. Recently, scoring balloon has presented an interventional tool for better plaque modification and improved stent or balloon expansion in lesion preparation. Nonslip element (NSE) balloon is a novel scoring balloon as used in Nishiyama et al. (17). It is similar to cutting balloon and can partially prevent elastic recoil and limit traumatic vascular wall injury. According to the recent study (25), NSE balloon was proved safe and effective in the treatment of *de novo* CAD combined with DCB. The clinical outcomes including CD, MI, and TLR and the angiographic outcomes including LLL, MLD1, and MLD2 were brought to assess the efficacy and safety of DCB. In this study, CD could be explained as death caused by cardiac factors and MI was defined by the latest ESC guidelines (10)_._ TLR was defined as any repeat revascularization due to restenosis in treated segments including 5 mm proximally and distally. We retrospected the included study and found the definitions of clinical outcomes in each study met our criteria. For angiographic outcomes, both LLL and MLD were defined and measured by QCA according to the included study (17–19). From the eligible studies included in this meta-analysis, only Her et al. (20) found CD events, and MI events were found in Gobic et al. studies (18). Thus, the subgroup analysis of CD and MI was abandoned because of studies which is not enough. For TLR, there was no significant difference between the DCB and stent group in RCT and OS. No significant difference was observed in subgroup analysis either. The precise quantification of luminal narrowing had been a challenge for a long time before, and it was solved since percutaneous transluminal coronary angioplasty (PTCA) by Andreas Grüntzig in 1977 was generated, which was also the greatest incentive to the development of QCA. QCA has played an important part in evaluating interventional techniques and assessing the results of DCB and stents (26, 27). QCA metrics including LLL and MLD (including MLD1 and MLD2) were chosen as angiographic outcomes in this study. Since Her et al. (20) did not provide angiographic outcomes, the results of OS were excluded as only 1 OS could not be calculated the CES and not convincing enough. Thus, 3 RCTs (17–19) were included to explore the angiographic outcomes_._ For MLD1, it could be observed that MLD1 was smaller in DCB group than stent group in RCT. It presented similar results in MLD2. The subgroup analysis showed that both MLD1 and MLD2 were smaller in each subgroup. MLD in this study was at postprocedural and follow-up, and it should be considered with the preprocedural MLD together. We retrospected the included study but failed to obtain the data of preprocedural MLD. The reasons which were inferred might be that the operators would be more likely to prefer stents than DCB when the target lesions were larger or might be attributed to the treatment allocation itself. LLL was introduced to assess the real changes for the lesions. For LLL, it could be regarded as a mark of unfavorable remodeling, and the mechanism might be inferred as an increase in the plaque area (28). The results showed no significant difference between DCB and stents with significant heterogeneity in RCT. Although the heterogeneity was lower (*I*^2^ from 90% turned to 58%) when the follow-up was more than 6 months according to the subgroup analysis, it still remained a moderate heterogeneity and it was not able to determine the source of heterogeneity.

As the most widely used procedure of PCI, stents are unceasing, updating, and developing. BMS has improved the cardiac hemodynamics though there are a series of complications, such as in-stent restenosis (ISR). The first-generation DES, with antiproliferative drugs such as paclitaxel or sirolimus, was investigated and reduced the rate of ISR compared with BMS. However, the late and very late stent thrombosis still exists in the first-generation DES. The dual antiplatelet therapy will also increase the risk of bleeding. To overcome these complications, the second- or newer-generation DES has been investigated with improved stent platforms, biocompatible, durable, or biodegradable polymers, and newer antiproliferative agents, even new technologies, such as ultrathin strut and polymer-free DES, which were developed and assessed by several trials. Studies showed that the risk of early or late complications was reduced in ACS and showed a better effect in patients with diabetes. Besides, it allowed shorter dual antiplatelet therapy in patients at high risk of bleeding, avoiding bleeding complications. It could also be applied in more complex lesions, such as left main artery lesions, bifurcation, dissection, and calcification with the help of other devices (29–31). There were also some limitations of DES as mentioned before, and more studies on the newer-generation DES were needed to assess the efficacy and safety. DCB has been gradually applied in clinical, which consists of 3 parts: balloon, antiproliferative drug, and drug carrier (coating). It attaches a layer of drug that can inhibit cell proliferation on the surface of the balloon. When the balloon expands, the drug will be fully contacted with the intima of the coronary artery stenosis in a short time and absorbed by the coronary artery tissue. With the development of research, the technology of the drug coating, balloon, and other aspects have been explored further, so as to improve the biocompatibility of balloon, the antirestenosis effect of coating optimize the control of drug time and release rate to reduce the risk of thrombosis (11–13). In the aspect of antiproliferative drugs, paclitaxel, as a cytotoxic drug, can block mitosis and inhibit the proliferation of vascular smooth muscle cells. It is also highly lipophilic and spreads to the vascular wall easily when contacting with intima, achieving the treatment of vascular stenosis. In clinical practice, paclitaxel-coated balloon (PCB) shows the acute surgical effect, reduces the incidence of related complications, and has a high level of immediate technical performance, sufficient short-term efficacy and safety (32). In addition to paclitaxel, sirolimus and other rapamycin derivatives as a cytotoxic drug can also be used in drug balloons, and relevant trials have proved that sirolimus-coated balloon is safe, with a lower incidence of adverse events and the rate of vascular reconstruction (33, 34).

## Limitations

There are also some limitations to our study. First, the number of studies we included is relatively small and the sample size is not large enough, which limited the power to detect the difference between DCB and stent group. Second, it still remains heterogeneity in the results of LLL and the origin of heterogeneity is still unknown though subgroup analysis was carried. It is difficult to discuss the source of heterogeneity with quantitative analysis such as meta-regression limited by the number of included studies. The publication bias had not been carried out since the studies included were less than 10. Third, some major adverse clinical events such as stent thrombosis and major bleeding were not added to this study because of the limited number of studies. Besides, the specific information on concomitant medication and risk factors were not reported in the included studies, and thus, it could not be analyzed for further evaluation. Finally, the difference among DCB technologies fails to analyze since there were limited data on new-generation sirolimus DCB. In this study, the antiproliferative drugs were all paclitaxel though the DCB brands were different. More RCT about new-generation sirolimus DCB are needed to be carried out.

## Conclusion

This systematic review and meta-analysis of RCT showed that DCB might provide a promising way on *de novo* non-small CAD compared with stents. However, more RCTs are still needed to further prove the benefits of the DCB strategy.

## Data Availability Statement

The original contributions presented in the study are included in the article/supplementary material, further inquiries can be directed to the corresponding author.

## Author Contributions

All authors contributed to the study's conception and design. Material preparation, data collection, and analysis were performed by KS and ZL. HW would solve the disagreements between KS and ZL. The first draft of the manuscript was written by KS and all authors commented on previous versions of the manuscript. All authors read and approved the final manuscript.

## Conflict of Interest

The authors declare that the research was conducted in the absence of any commercial or financial relationships that could be construed as a potential conflict of interest.

## Publisher's Note

All claims expressed in this article are solely those of the authors and do not necessarily represent those of their affiliated organizations, or those of the publisher, the editors and the reviewers. Any product that may be evaluated in this article, or claim that may be made by its manufacturer, is not guaranteed or endorsed by the publisher.

## References

[1] Khan MA Hashim MJ Mustafa H Baniyas MY Al Suwaidi SKBM AlKatheeri R . Global epidemiology of ischemic heart disease: results from the global burden of disease study. Cureus. (2020) 12:e9349. 10.7759/cureus.9349 32742886 PMC7384703

[2] Hoole SP Bambrough P. Recent advances in percutaneous coronary intervention. Heart. (2020) 106:1380–6. 10.1136/heartjnl-2019-315707 32522821

[3] Li SM Li YL Wang SQ Bai CC. Progress in the processing technology of coronary stents with micro/nano structures. OPTIK. (2017) 2017:319–24. 10.1016/j.ijleo.2017.08.136

[4] Madhavan MV Stone GW. Adverse events beyond 1 year after percutaneous coronary intervention. Curr Opin Cardiol. (2020) 35:687–96. 10.1097/HCO.0000000000000792 32852348

[5] Sharifi Z Yazdi MJ Eshraghi A Vakili V Ramezani J. Clinical outcomes and complications of treatment with supraflex stent in patients with coronary artery disease: one-year follow-up. Eur J Transl Myol. (2019) 29:8231. 10.4081/ejtm.2019.8231 31354927 PMC6615074

[6] Nestelberger T Kaiser C Jeger R. Drug-coated balloons in cardiovascular disease: benefits, challenges, and clinical applications. Expert Opin Drug Deliv. (2020) 17:201–11. 10.1080/17425247.2020.1714590 31918593

[7] Shibutani H Akita Y Oishi Y Sueyoshi H Mukai Y Yutaka K . The potential hazard of a non-slip element balloon causing distal longitudinal stent deformation: the first clinical experience and in vitro assessment. Cardiol J. (2019) 26:645–52. 10.5603/CJ.a2018.0065 29924377 PMC8083032

[8] Hemu M Kalra D. Coronary artery pseudoaneurysm: a rare complication of drug-eluting stenting. J Am Coll Cardiol. (2019) 73:2274. 10.1016/S0735-1097(19)32880-3

[9] Riku S Suzuki S Jinno Y Tanaka A Ishii H Murohara T. Coronary drug-eluting stent infection complicated by coronary artery aneurysm and purulent pericarditis: complete resolution without surgery. Can J Cardiol. (2020) 36:967.e1–967.e3. 10.1016/j.cjca.2020.01.007 32407676

[10] Neumann FJ Sousa-uva M Ahlsson A Alfonso F Banning AP Benedetto U . 2018 ESC/EACTS Guidelines on myocardial revascularization. Euro Intervention. (2019) 14:1435–534. 10.4244/EIJY19M01_01 30667361

[11] Jeger RV Eccleshall S Wan Ahmad WA Ge J Poerner TC Shin ES . Drug-coated balloons for coronary artery disease: third report of the international DCB consensus group. JACC Cardiovasc Interv. (2020) 13:1391–402. 10.1016/j.jcin.2020.02.043 32473887

[12] Gao L Chen YD. Application of drug-coated balloon in coronary artery intervention: challenges and opportunities. J Geriatr Cardiol. (2016) 13:906–13. 10.11909/j.issn.1671-5411.2016.11.005 28133467 PMC5253407

[13] Meneguz-Moreno RA Ribamar Costa J Jr Abizaid A. Drug-coated balloons: hope or hot air: update on the role of coronary DCB. Curr Cardiol Rep. (2018) 20:100. 10.1007/s11886-018-1025-4 30171374

[14] Megaly M Rofael M Saad M Rezq A Kohl LP Kalra A . Outcomes with drug-coated balloons in small-vessel coronary artery disease. Catheter Cardiovasc Interv. (2019) 93:E277–86. 10.1002/ccd.27996 30489687

[15] Liu W Zhang M Chen G Li Z Wei F. Drug-coated balloon for *de novo* coronary artery lesions: a systematic review and trial sequential meta-analysis of randomized controlled trials. Cardiovasc Ther. (2020) 2020:4158363. 10.1155/2020/4158363 32934664 PMC7482020

[16] Li M Guo C Lv YH Zhang MB Wang ZL. Drug-coated balloon versus drug-eluting stent in *de novo* small coronary vessel disease. Medicine (Baltimore). (2019) 98:e15622. 10.1097/MD.0000000000015622 31124941 PMC6571399

[17] Nishiyama N Komatsu T Kuroyanagi T Fujikake A Komatsu S Nakamura H . Clinical value of drug-coated balloon angioplasty for *de novo* lesions in patients with coronary artery disease. Int J Cardiol. (2016) 222:113–8. 10.1016/j.ijcard.2016.07.156 27494722

[18] Gobić D Tomulić V Lulić D Židan D Brusich S Jakljević T . Drug-coated balloon versus drug-eluting stent in primary percutaneous coronary intervention: a feasibility study. Am J Med Sci. (2017) 354:553–60. 10.1016/j.amjms.2017.07.005 29208251

[19] Vos NS Fagel ND Amoroso G Herrman JR Patterson MS Piers LH . Paclitaxel-coated balloon angioplasty versus drug-eluting stent in acute myocardial infarction. JACC Cardiovasc Interv. (2019) 12:1691–9. 10.1016/j.jcin.2019.04.016 31126887

[20] Her AY Kim YH Garg S Shin ES. Impact of paclitaxel-coated balloon versus newer-generation drug-eluting stent on periprocedural myocardial infarction in stable angina patients. Coron Artery Dis. (2018) 29:403–8. 10.1097/MCA.0000000000000620 29620557

[21] Iwasaki Y Koike J Ko T Funatsu A Kobayashi T Ikeda T . Comparison of drug-eluting stents vs. drug-coated balloon after rotational atherectomy for severely calcified lesions of non-small vessels. Heart Vessels. (2021) 36:189–99. 10.1007/s00380-020-01684-z 32857188

[22] Lin Y Sun X Liu H Pang X Dong S. Drug-coated balloon versus drug-eluting stent for treating *de novo* coronary lesions in large vessels: a meta-analysis of clinical trials. Herz. (2020) 46:269–76. 10.1007/s00059-020-04938-8 32468141

[23] Moher D Liberati A Tetzlaff J Altman DG PRISMA Group. Preferred reporting items for systematic reviews and meta-analyses: the PRISMA statement. J Clin Epidemiol. (2009) 62:1006–12. 10.1016/j.jclinepi.2009.06.005 19631508

[24] Cortese B. The role of optimal lesion preparation for de-novo coronary vessels when a stentless intervention strategy is planned. Minerva Cardioangiol. (2020) 68:51–6. 10.23736/S0026-4725.19.05091-6 31789010

[25] Bonaventura K Schwefer M Yusof AKM Waliszewski M Krackhardt F Steen P . Systematic scoring balloon lesion preparation for drug-coated balloon angioplasty in clinical routine: results of the PASSWORD observational study. Adv Ther. (2020) 37:2210–23. 10.1007/s12325-020-01320-2 32274746 PMC7467461

[26] Bax AM van Rosendael AR Ma X van den Hoogen IJ Gianni U Tantawy SW . Comparative differences in the atherosclerotic disease burden between the epicardial coronary arteries: quantitative plaque analysis on coronary computed tomography angiography. Eur Heart J Cardiovasc Imaging. (2021) 22:322–30. 10.1093/ehjci/jeaa275 33215192

[27] Collet C Grundeken MJ Asano T Onuma Y Wijns W Serruys PW. State of the art: coronary angiography. EuroIntervention. (2017) 13:634–43. 10.4244/EIJ-D-17-00465 28844026

[28] Yamamoto T Sawada T Uzu K Takaya T Kawai H Yasaka Y. Possible mechanism of late lumen enlargement after treatment for *de novo* coronary lesions with drug-coated balloon. Int J Cardiol. (2020) 321:30–7. 10.1016/j.ijcard.2020.07.028 32710988

[29] Azad MAS Gul U Javed A. Everolimus-eluting bioresorbable coronary scaffold in Asian patients: a 2-year follow-up. J Pak Med Assoc. (2020) 70:2281–4. 10.47391/JPMA.1149 33475614

[30] Kuramitsu S Sonoda S Ando K Otake H Natsuaki M Anai R . Drug-eluting stent thrombosis: current and future perspectives. Cardiovasc Interv Ther. (2021) 36:158–68. 10.1007/s12928-021-00754-x 33439454

[31] Spione F Brugaletta S. Second generation drug-eluting stents: a focus on safety and efficacy of current devices. Expert Rev Cardiovasc Ther. (2021) 19:107–27. 10.1080/14779072.2021.1874352 33417509

[32] Zhang D Yang R Wang S Dong Z. Paclitaxel: new uses for an old drug. Drug Des Devel Ther. (2014) 8:279–84. 10.2147/DDDT.S56801 24591817 PMC3934593

[33] Alfonso F Avanzas P. Sirolimus-coated balloons: ready for primetime in real world patients? J Cardiovasc Med. (2021) 22:101–3. 10.2459/JCM.0000000000001072 32858627

[34] Basavarajaiah S Athukorala S Kalogeras K Panoulas V Loku Waduge BH Bhatia G . Mid-term clinical outcomes from use of Sirolimus coated balloon in coronary intervention; data from real world population. Catheter Cardiovasc Interv. (2020). 10.1002/ccd.28998 32473075

